# Supplementary material for: Exposure of Cultured Hippocampal Neurons to the Mitochondrial Uncoupler Carbonyl Cyanide Chlorophenylhydrazone Induces a Rapid Growth of Dendritic Processes
Source: Int J Mol Sci. 2023 Aug 18;24(16):12940. doi: 10.3390/ijms241612940 (PMC10455170; doi:10.3390/ijms241612940)
Supplement: Supplementary file 1 [file ijms-24-12940-s001.zip › CCCP new figures.videos/Supplementary Videos.pdf]

Supplementary Material:

Supplementary Video S1. Effect of CCCP on the morphology of hippocampal neuron. Continuous time lapse imaging of morphological changes in eBFP-transfected neuron in the presence of CCCP was performed during about 31 min (pre-incubation with CCCP 10 minutes, not shown, to avoid bleaching) at the rate of 0.33 frame/s. In the video file, 1 min = 1 s. Frame size is about 211 × 74 μm. Note new protrusion appearance at 10th min video (20th min with CCCP) and first signs of dendrite blabbing at about 20th min (30th min with CCCP).

Supplementary Video S2. Effect of CCCP on new protrusion outgrowth and mitochondrial calcium rise in a dendritic segment of hippocampal neuron. Neuron was co-transfected with eBFP (morphology, cyan) and mtRCaMP (mitochondrial calcium, red). The video represents recording during about 37 min. In the video file, 3 min = 1 s. The frame size is about 93 × 9.5 μm. Note new protrusion appearance and influx of calcium into mitochondria at about 18th min of recording.

Supplementary Video S3. Sequential calcium influx into mitochondria, stretched along the dendrite of hippocampal neuron. The cell was transfected with mitochondrial calcium-sensitive protein mtRCaMP. Cytosolic calcium indicator Fluo-2AM was loaded prior to imaging. Video recording was performed during 30 min. In the video file, 1 min = 1 s. The frame size is about 145 × 19.5 μm. Top frame, Fluo-2 fluorescence, green. Bottom frame, mtRCaMP fluorescence, red. Note the beginning of cytosolic calcium rise at about 12th min and the initiation of calcium influx into mitochondria starting from the left to the right of the frame at about 19th min.
