# Supplementary material for: Exposure of Cultured Hippocampal Neurons to the Mitochondrial Uncoupler Carbonyl Cyanide Chlorophenylhydrazone Induces a Rapid Growth of Dendritic Processes
Source: Int J Mol Sci. 2023 Aug 18;24(16):12940. doi: 10.3390/ijms241612940 (PMC10455170; doi:10.3390/ijms241612940)
Supplement: Supplementary file 1 [file ijms-24-12940-s001.zip › SUPPLEMENTARY FIGURES.pdf]

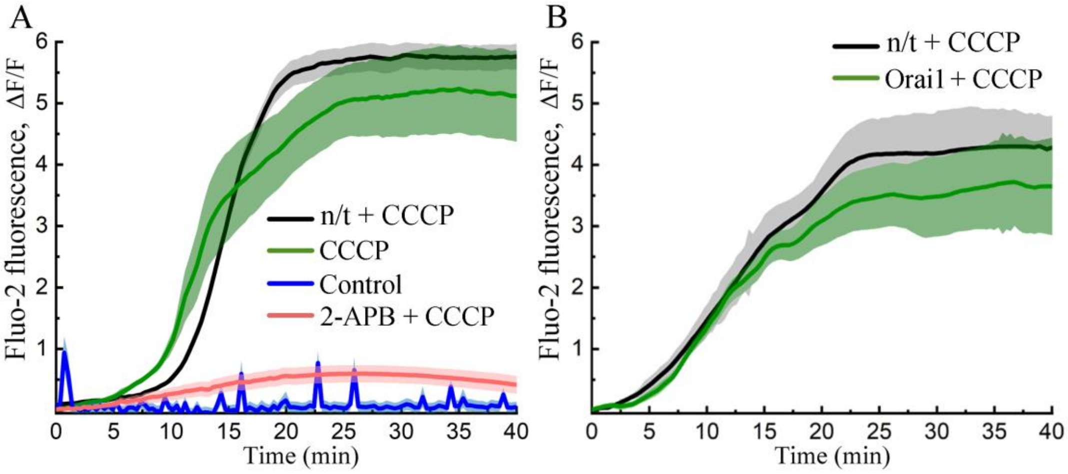

Supplementary Figure S1. Transfection does not affect [Ca^{2+}]i in control and CCCP treatment. A. The effect of 2-APB (n = 16 cells, carrot curve) on [Ca^{2+}]i following CCCP. Control transfected cells (**e**BFP) with CCCP: n = 15 cells, green curve; control non-transfected (n/t) cells with CCCP: n = 20 cells, black curve; blue: non-transfected untreated cells: n = 35 cells. Data are presented as mean ± SEM (shadows) from three cell cultures, DIV 10-14. Note that in the absence of CCCP, control cells (blue) express spontaneous calcium transients. **B.** Cells transfected with **e**BFP, Orai1 and with cytosolic calcium indicator (green curve, n = 15) and n/t cells in the same field (black curve, n=16) with CCCP treatment.

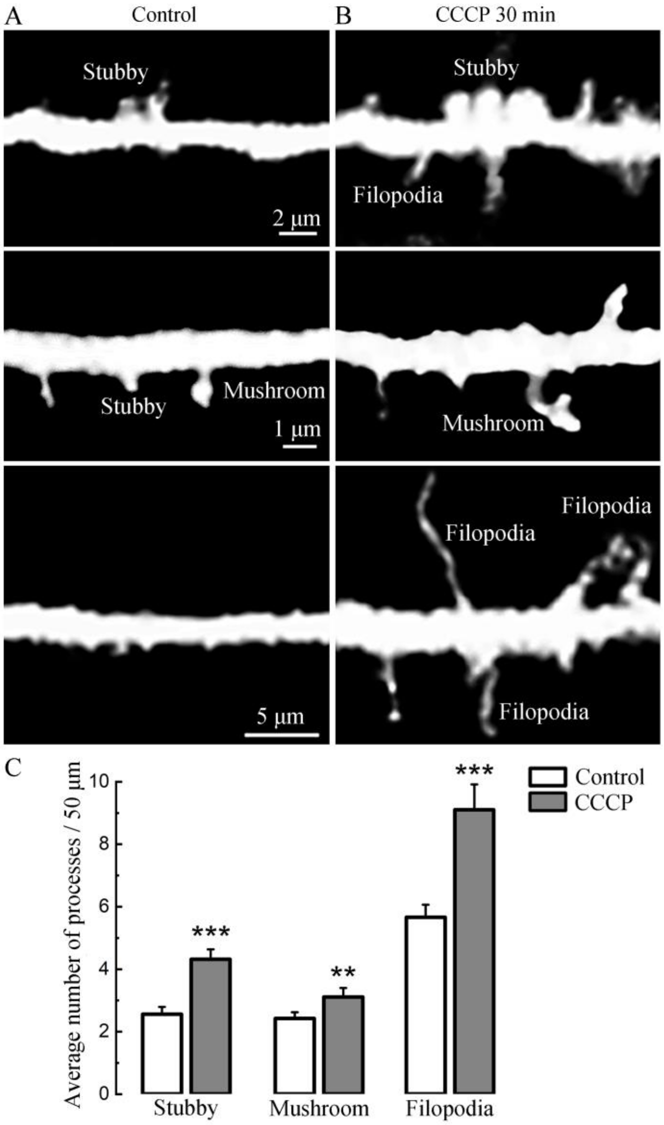

Supplementary Figure S2. A&B. Examples of dendritic areas transfected with eBFP (monochrome) in control (**A**) and after 30 minutes with CCCP treatment (**B**). **C.** Averages number of dendritic processes (filopodia, mushroom and stubby spines) detected with eBFP: control stubby / 30 min CCCP stubby 2.55 ± 0.23 / 4.23 ± 0.3 ***, $p < 0.001$; control mushroom spines / 30 min CCCP mushroom spines 2.48 ± 0.22 / 3.32 ± 0.27 **, $p < 0.01$; control filopodia / 30 min CCCP filopodia 5.57 ± 0.37 / 8.34 ± 0.69 ***, $p < 0.001$; n = 20 cells from four cell cultures 10-14 DIV, a comparable number of dendritic sections, 50 μm long, were analyzed for each cell, *t*-tests.

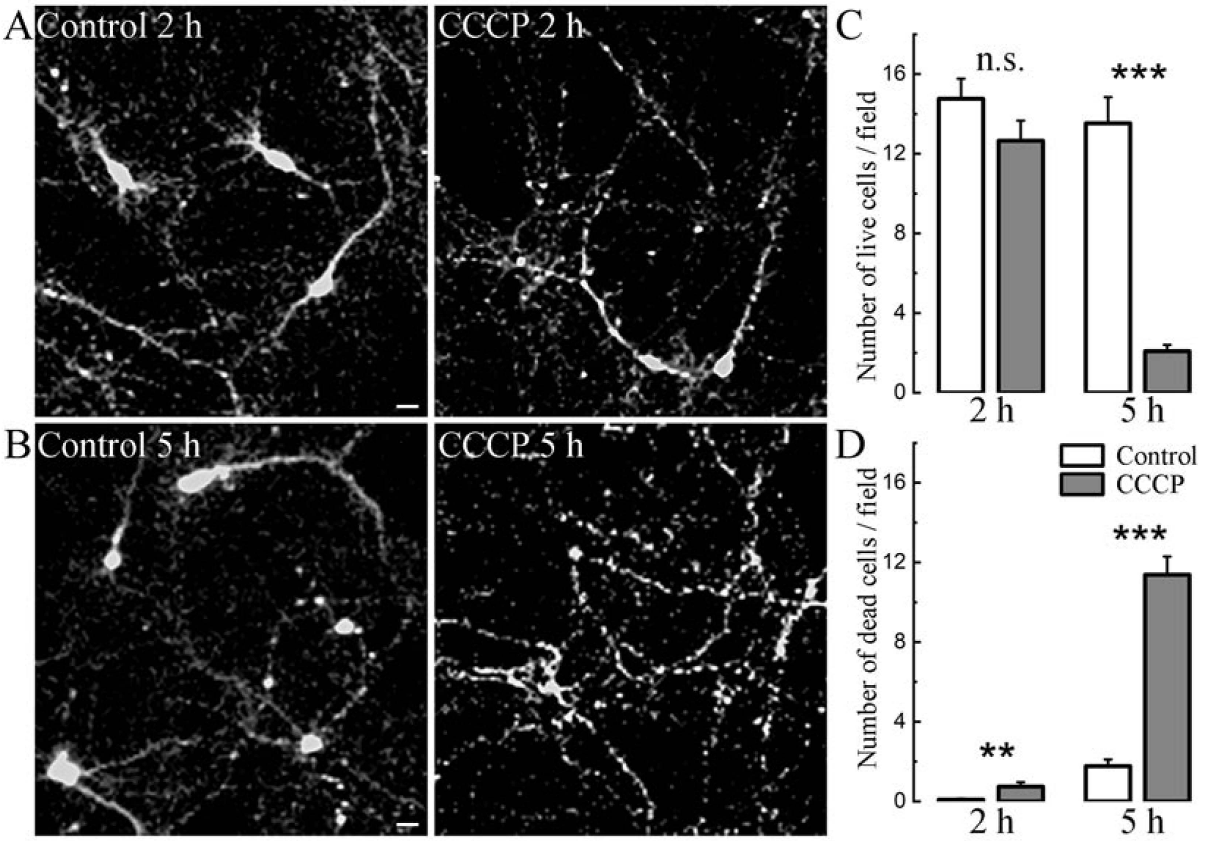

Supplementary Figure S3. A&B. imaging in CCCP-containing medium, EGFP (Scare bar 10 μm). **C.** Number of living cells in control medium (n = 34 fields / 17 fields) and with CCCP (n = 35 fields / 24 fields) after 2 and 5 hours of imaging. Control 2 h /CCCP 2 h: n.s.; Control 5 h / CCCP 5 h: ***, $p < 0.001$. **D.** Number of dead cells in control medium and with CCCP after 2 and 5 hours of imaging. Control 2 h / CCCP 2 h: **, $p < 0.01$; Control 5 h / CCCP 5 h: ***, $p < 0.001$. Same fields for **C** and **D**, two cell cultures, *t*-tests.
